# Intraoperative Lateral Hinge Fractures and Early Postoperative Pain Burden After Medial Opening Wedge High Tibial Osteotomy: Implications for Augmented Postoperative Analgesia

**DOI:** 10.7759/cureus.115457

**Published:** 2026-08-30

**Authors:** Woon-Hwa Jung, Yogendra Agiwal, Saksham Goyal, Dong-Hyun Kim, Ryohei Takeuchi

**Affiliations:** 1 Orthopedics, Murup Hospital, Changwon, KOR; 2 Orthopedics, Saiwai Tsurumi Hospital, Yokohama, JPN

**Keywords:** knee osteoarthritis, lateral hinge fracture, medial open wedge high tibial osteotomy, multimodal analgesia, postoperative pain intensity, visual analog scale

## Abstract

Background

Intraoperative lateral hinge fractures (LHFs) are common complications during medial opening wedge high tibial osteotomy (MOWHTO). While their radiological outcomes are well-studied, their impact on immediate postoperative pain remains underexplored.

Purpose

The aim of this study was to evaluate the relationship between LHFs and early postoperative pain intensity (visual analog scale [VAS] scores) following MOWHTO.

Materials and methods

We retrospectively reviewed 244 patients undergoing MOWHTO under spinal anesthesia. Patients were divided into a hinge fracture group (n = 95) and a non-hinge fracture group (n = 149). Fractures were classified using a novel MRI-based system. Preoperative pain sensitivity was balanced using needle-prick antibiotic sensitivity testing (AST) VAS scores. Postoperative day 1 (POD#1) VAS scores and analgesic use were compared under a standardized multimodal analgesic protocol.

Results

Baseline demographics and preoperative AST pain sensitivity were similar between groups (p = 0.1293). On POD#1, the hinge fracture group experienced significantly higher mean pain intensity than the non-fracture group (mean VAS: 3.82±1.97 vs. 3.30±1.85; p = 0.0411) and trended toward higher rates of moderate-to-severe pain (31.6% vs. 19.5%), though this categorical distribution did not achieve strict statistical significance (χ² = 4.95, p = 0.084). Importantly, multivariable regression confirmed that sustaining a hinge fracture remained an independent predictor of elevated pain regardless of age, sex, or BMI (β = 0.53, p = 0.036). Fracture morphology did not significantly influence pain levels (p = 0.434). Total analgesic consumption was uniform across both cohorts (p = 0.9981)

Conclusion

While intraoperative LHFs are associated with a statistically significant increase in early postoperative pain following MOWHTO, the mean difference (0.52 points) does not meet the commonly accepted minimal clinically important difference. This indicates that a rigorous, standardized multimodal analgesic protocol effectively neutralizes the excess nociceptive burden associated with the fracture. Clinicians utilizing robust multimodal pain pathways can proceed with early rehabilitation protocols without the need to drastically alter their postoperative management when an LHF occurs.

## Introduction

Medial compartment osteoarthritis (OA) of the knee is a prevalent degenerative condition, often causing chronic pain and functional impairment in middle-aged, active patients. It is characterized by varus deformity that initiates a self-perpetuating cycle of increased medial joint loading and progressive cartilage degradation [1,2]. Medial opening wedge high tibial osteotomy (MOWHTO) has emerged as a highly successful, joint-preserving surgical intervention for patients suffering from medial compartment knee OA. By realigning the mechanical axis of the lower limb, the procedure effectively redistributes weight-bearing forces, thereby delaying the need for more invasive arthroplasty [3,4]. While clinical and radiological outcomes following MOWHTO are generally highly favorable, the procedure is not without its technical challenges. Among these, the occurrence of intraoperative lateral hinge fractures (LHFs) remains a notable and frequently encountered complication. Such fractures can disrupt the structural integrity of the lateral cortex, potentially complicate the biomechanical stability of the osteotomy site, and influence the subsequent postoperative recovery [5,6].

Effective pain management in the immediate postoperative period is widely recognized as a critical determinant of successful surgical recovery, facilitating early mobilization, improving patient satisfaction, and reducing the risk of postoperative complications [7]. Despite the prevalence of LHFs, the direct impact of this intraoperative injury on immediate postoperative pain intensity remains underexplored in orthopedic literature. Understanding this relationship is essential, as elevated pain levels may pose a significant barrier to the aggressive rehabilitation protocols required for optimal MOWHTO outcomes.

The primary objective of this study was to evaluate the impact of intraoperative LHFs on early postoperative pain intensity (visual analog scale [VAS] scores) following MOWHTO. We hypothesized that patients who sustain a hinge fracture experience significantly higher postoperative pain than those without fractures. The secondary objective was to assess whether a standardized institutional multimodal analgesic protocol could effectively manage this additional nociceptive burden without necessitating an increase in total postoperative analgesic consumption.

## Materials and methods

Study design and data collection

A retrospective evaluation was conducted on patients undergoing MOWHTO from January 2019 to April 2025 to evaluate the impact of intraoperative LHFs on postoperative pain. The primary outcome measure was the intensity of immediate postoperative pain the day after surgery, evaluated using the VAS. An initial cohort of 254 patients was evaluated. Ten patients were subsequently excluded from the analysis: eight who received general anesthesia and two in whom the effects of spinal anesthesia had not fully subsided. Consequently, a total of 244 patients were included in the final analysis. The cohort was stratified into two groups: 95 patients (38.9%) who sustained an intraoperative hinge fracture (hinge fracture group) and 149 patients (61.1%) who did not (non-hinge fracture group). Pain scores were compared between the two groups. Additionally, patients were categorized by pain severity: minimal pain (VAS 0-2), mild pain (VAS 3-4), and moderate-to-severe pain (VAS 5-10). Preoperative baseline pain sensitivity was assessed using the needle-prick VAS score, obtained during routine preoperative antibiotic sensitivity testing (AST).

Assessment and fracture classification

To precisely evaluate the presence and type of fracture, we utilized the novel magnetic resonance imaging (MRI)-based classification system recently developed and published by the senior authors of the present study (Jung et al.) [8]. According to it, intraoperative LHFs were systematically categorized into four distinct subtypes on the basis of the fracture line pattern: type A (proximal to the tibiofibular joint), type B (into the proximal tibiofibular joint), type C (distal to the tibiofibular joint), and type D (proximal into the joint).

Postoperative analgesic protocol

All patients were managed postoperatively using a standardized, institutional multimodal analgesic protocol. Upon completion of surgery, all patients were initiated on an intravenous patient-controlled analgesia (IV PCA) pump. The PCA contained fentanyl at a concentration of 10 mcg/mL, programmed with a continuous background infusion of 1 mL/hour (10 mcg/hour), a demand bolus of 1.5 mL (15 mcg), and a lockout interval of 15 minutes.

The administration of rescue analgesia was strictly protocolized and driven entirely by the patient’s reported pain scores, removing treating clinician discretion as a potential confounder. Ward nursing staff - who were not involved in the surgical procedure and were blinded to the intraoperative fracture status - administered step-up rescue medications based on the following specific thresholds:

All patients received IV diclofenac or acetaminophen (1 ampoule) routinely postoperatively as baseline non-opioid coverage.

For patients reporting moderate-to-severe breakthrough pain (VAS ≥ 5), intramuscular (IM) tramadol was administered.

If severe pain (VAS ≥ 7) persisted despite tramadol, IM pethidine (50 mg/mL) was utilized.

Patients with preserved renal function were eligible for an additional IV infusion of diclofenac (2 ampoules in 1L of fluid) between opioid doses if moderate pain persisted. Because this step-up protocol was strictly dictated by real-time VAS thresholds rather than physician discretion, treatment bias regarding the patient's fracture status was effectively eliminated.

A sedative was available between 9:00 PM and 1:00 AM for patients with stable vital signs.

To provide sustained background analgesia, transdermal patches (fentanyl 12 mcg/hour or buprenorphine 5 mcg/hour) were applied on the morning of postoperative day (POD) #1 at 8:00 AM. Crucially, the primary outcome measure - the POD#1 VAS score - was recorded by blinded nursing staff at 7:00 AM during morning rounds, prior to the application of the transdermal patch, ensuring that the patch did not confound the immediate postoperative pain assessment.

Statistical analysis

All statistical analyses were performed using SPSS version 26.0 (IBM Corp., Armonk, NY), and a two-tailed p-value of less than 0.05 was considered statistically significant. Continuous variables were expressed as mean ± standard deviation or median with interquartile range (IQR), as appropriate. To compare baseline demographic data, the independent Student's t-test was used for continuous variables, and the chi-square test was applied to categorical variables (gender and surgical side). Normality of the data was assessed using the Shapiro-Wilk test. Because the postoperative VAS scores were non-normally distributed (p < 0.001), a non-parametric Mann-Whitney U test was implemented to evaluate differences in pain intensity (POD#1 VAS) between the two groups, cross-validated with the independent Welch t-test. The Mann-Whitney U test was also employed to compare preoperative pain sensitivity (AST VAS) and total analgesic consumption, as these data were also non-normally distributed. Individual analgesic medication use (categorical) was analyzed using the chi-square test or Fisher’s exact test. Finally, for subgroup analysis restricting evaluation to the fracture cohort, the Kruskal-Wallis test was performed to compare pain scores across fracture classifications (types A, B, and C). Type D fractures were excluded from this specific statistical plan, as zero instances of this subtype were observed within our study cohort.

## Results

Patient demographics and baseline characteristics

A total of 244 patients were included in this study, comprising 95 patients in the hinge fracture group and 149 patients in the non-fracture group. The cohorts were well-matched regarding mean age (p = 0.218, t = 1.23), mean BMI (p = 0.153, t = 1.44), gender (p = 0.361, χ² = 0.83), and surgical side distribution (p = 0.412, χ² = 0.67) (Table 1).

**Table 1:** Patient demographics and baseline characteristics BMI, body mass index; SD, standard deviation; t, Student's t-test statistic; χ², chi-square test statistic

Variable	Hinge Fracture Group (95 Patients)	Non-Hinge Fracture Group (149 Patients)	Statistical Test and Value	p-Value
Age (years, mean ± SD)	58.23 ± 6.72	59.35 ± 7.05	t = 1.23	0.2179
BMI (mean ± SD)	26.37 ± 4.18	25.63 ± 3.41	t = 1.44	0.1525
Gender (female/male)	59/36	103/46	χ² = 0.83	0.361
Surgical side (left/right)	47/48	83/66	χ²= 0.67	0.4124

Preoperative baseline sensitivity (AST VAS)

We compared preoperative pain sensitivity - measured via the needle-prick VAS score obtained during routine preoperative AST - to ensure that both groups maintained an equivalent baseline. The hinge fracture group reported a mean score of 4.07, while the non-fracture group reported a mean of 3.73. The Mann-Whitney U test indicated no statistically significant difference between the two groups (p = 0.1293, U = 6612.5), confirming that both cohorts had similar preoperative baseline pain sensitivity.

Postoperative pain outcomes

Primary outcome analysis revealed that the hinge fracture group experienced significantly higher postoperative pain intensity compared to the non-hinge fracture group. The hinge fracture group demonstrated a statistically higher mean VAS score (3.82 ± 1.97) compared to the non-hinge fracture group (3.30 ± 1.85). This difference was confirmed by both the independent Welch’s t-test (t = 2.05, p = 0.041) and the non-parametric Mann-Whitney U test (U = 6012.50, p = 0.038) (Table 2). Furthermore, patients in the hinge fracture group trended toward higher rates of moderate-to-severe pain (31.6% vs. 19.5%) and lower rates of minimal pain (28.4% vs. 36.9%) than those in the non-fracture group. While this specific categorical distribution of pain severity did not reach statistical significance (χ² = 4.95, p = 0.084), multivariable linear regression analysis demonstrated that the presence of a hinge fracture remained an independent predictor of higher POD#1 VAS scores after adjusting for baseline covariates including age, sex, and BMI (β = 0.53, p = 0.036). Finally, for subgroup analysis restricting evaluation to the fracture cohort, the Kruskal-Wallis test was performed to compare pain scores across fracture classifications (types A, B, and C). Notably, no type D (intra-articular) fractures occurred in our sample, and thus this subtype was not represented in the analysis. Regarding fracture morphology, there was no statistically significant difference in POD#1 VAS scores across the different fracture types (type A: 3.50 ± 2.00; type B: 4.02 ± 1.84; type C: 3.82 ± 2.30; p = 0.434, χ² = 1.67) (Table 3).

**Table 2:** Clinical pain outcomes (POD#1) IQR, interquartile range; POD#1, postoperative day 1; SD, standard deviation; t, Student's t-test statistic; U, Mann-Whitney U test statistic; VAS, visual analog scale

Variable	Hinge Fracture (n = 95)	Non-Fracture (n = 149)	Statistical Test and Value	p-Value
VAS score, mean ± SD	3.82 ± 1.97	3.30 ± 1.85	t = 2.05	0.041
VAS score, median (IQR)	3.0 (2.0–5.0)	3.0 (2.0–4.0)	U = 6012.5	0.038

**Table 3:** Subgroup analysis of postoperative pain scores across lateral hinge fracture classifications Type D fractures (proximal into the joint) are included in the utilized classification system but do not appear in this table as zero cases were observed in the study cohort. IQR, interquartile range; SD, standard deviation; VAS, visual analog scale; χ², chi-square test statistic (Kruskal-Wallis)

Fracture Subtype	Number of Patients, n	VAS Score, Mean ± SD	VAS Score, Median (IQR)	Statistical Test and Value	p-Value
Type A	30	3.50 ± 2.00	3.0 (2.0–4.5)	χ² = 1.67	0.434
Type B	48	4.02 ± 1.84	4.0 (3.0–5.0)	(Kruskal-Wallis)	-
Type C	17	3.82 ± 2.30	3.5 (2.0–5.0)	-	-

Analgesic utilization

To assess the consistency of our postoperative pain management, “total analgesic consumption” was quantified by analyzing the frequency of supplemental rescue analgesic administrations (total number of PRN doses of tramadol, pethidine, or additional diclofenac) required beyond the baseline PCA. PCA utilization itself was identical between groups based on standard continuous infusion rates. Both analyses yielded no statistically significant differences (p > 0.05, χ² = 0.54, for medication classes; p = 0.9981, t = 0.002 for total rescue analgesic requirement), demonstrating that the standardized analgesic protocol was applied with uniformity across the study population.

## Discussion

This study demonstrates that the occurrence of an intraoperative LHF significantly increases immediate postoperative pain intensity following MOWHTO, even when standardized institutional analgesic protocols are strictly maintained. These findings highlight that hinge fractures are not merely radiological complications but clinical occurrences that elevate the burden of early postoperative pain.

While the hinge fracture group demonstrated a statistically significant elevation in POD#1 VAS scores (p=0.041), this finding must be interpreted through a clinical lens. The absolute mean difference in VAS scores between the cohorts was 0.52 points. In acute pain assessment, the widely established minimal clinically important difference (MCID) for the 10-point VAS is commonly cited as approximately 1.0 to 1.4 points. Because our observed difference falls below this clinical threshold, the elevated pain, though statistically detectable, is likely imperceptible to the patient. This sub-MCID finding demonstrates that the standardized, institutional multimodal analgesic protocol (incorporating IV PCA, targeted NSAIDs, and opioids) is highly effective at blunting the heightened nociceptive drive caused by the structural disruption of the fracture.

Subgroup analysis revealed that pain intensity was independent of specific fracture morphology, suggesting that the occurrence of an intraoperative hinge fracture is the primary determinant of increased pain. This simplifies clinical decision-making, as surgeons should anticipate increased pain whenever a fracture is recognized, regardless of its type. Given the accelerated rehabilitation protocols typical of high tibial osteotomy, effective pain management is essential to facilitate timely mobilization. We utilized preoperative needle-prick AST VAS scores as a pragmatic attempt to estimate baseline pain sensitivity between the cohorts. While this novel metric suggested no significant baseline differences, the observed increase in postoperative pain is likely multifactorial, driven primarily by the fracture’s structural disruption of the highly innervated periosteum and the resulting exaggerated localized inflammatory response.

The heightened pain intensity observed in the hinge fracture group likely stems from a combination of mechanical and biological factors. A fracture involves a breach of the cortical bone and trauma to the periosteum, which is highly innervated by nociceptive fibers. This structural injury likely triggers an exaggerated localized inflammatory response. Because the fracture involves an additional breach of the highly innervated periosteum and lateral cortex, we hypothesize that the local release of nociceptive mediators, such as pro-inflammatory cytokines and prostaglandins, plausibly exceeds the inflammatory baseline of an uncomplicated osteotomy, providing a mechanical and biological rationale for the elevated pain levels [9]. Furthermore, despite internal fixation with a locking plate, the loss of the hinge structural integrity may introduce micro-instability at the osteotomy site. This motion, even if microscopic, can stimulate surrounding nociceptors, particularly during early postoperative mobilization. These findings highlight the importance of meticulous surgical technique to preserve the lateral hinge cortical integrity and ensure optimal stability.

While previous orthopedic research has extensively evaluated the impact of hinge fractures on bone healing, delayed union, and long-term radiological correction [5,10,11], very little data have quantified their impact on immediate postoperative pain. Ogawa et al. [12] evaluated the impact of LHFs following opening wedge distal tuberosity osteotomy (OWDTO). While OWDTO is a technical variant of standard MOWHTO designed to preserve patellar height, both procedures rely on a medial opening wedge and share an identical structural reliance on the lateral hinge. Consequently, the nociceptive response to a lateral cortical breach is highly comparable. In their cohort, Ogawa et al. found that patients sustaining an LHF experienced significantly higher pain levels during recovery. Specifically, these patients reported significantly higher pain at rest three weeks after surgery (p=0.043) and persistent, significantly higher pain during movement six months post-surgery (p<0.001) compared to those without hinge fractures. These findings regarding persistent pain are further corroborated by Jokar et al. [13], who similarly reported a significantly higher mean VAS score at the six-month follow-up among their LHF cohort (3.52 ± 0.67) compared to the non-LHF group (1.62 ± 1.77, p<0.001). These findings suggest that postoperative LHF contributes to increased pain burden that can extend into the mid-term follow-up. Consequently, while our data demonstrate the critical need for intensified analgesia in the immediate acute phase, combining our findings with existing literature suggests that pain management for patients with intraoperative hinge fractures should extend well beyond the first 24 hours to ensure that they can meet the demands of early rehabilitation.

Postoperative pain management for MOWHTO has increasingly shifted toward regional anesthesia techniques to optimize patient recovery while minimizing opioid reliance. Recent evidence highlights that ultrasound-guided regional blocks, such as the adductor canal block (ACB) and femoral nerve block, effectively reduce VAS pain scores in the immediate postoperative period [14,15]; notably, the ACB is often favored for providing significant analgesia without compromising quadriceps motor function. Furthermore, the efficacy of these peripheral nerve blocks can be substantially enhanced by incorporating adjuvants such as dexamethasone, which has been shown in systematic reviews to prolong the duration of sensory blockade and further decrease overall postoperative opioid consumption [16]. Another prospective randomized study reported that intraoperative periarticular multimodal drug injection in patients undergoing MOWHTO results in a significant reduction in VAS scores during the two-week postoperative period [17].

Given our findings, we propose that patients identified as having an intraoperative hinge fracture should be triaged for an augmented, targeted multimodal analgesic strategy. Rather than waiting for the manifestation of breakthrough pain, early intervention, such as preemptive regional nerve blocks or intensified local infiltration analgesia, could be tailored specifically for these patients to mitigate the increased pain caused by the fracture and improve patient comfort. Furthermore, recent evidence-based reviews on knee surgery analgesia highlight the necessity of scheduled, multimodal protocols over “as-needed” administration, particularly for patients with fracture-related nociceptive sensitization [18]. By targeting multiple pathways (e.g., prostaglandin inhibition via NSAIDs and central sensitization via non-opioids), clinicians can effectively dampen the movement-evoked pain that characteristically complicates the rehabilitation of patients with LHFs, thereby minimizing reliance on opioids and facilitating earlier functional outcomes [19].

This study has several limitations. First, its retrospective design restricts our ability to account for all psychological and individual factors that influence pain perception. Second, the small sample size of the fracture group likely underpowers the statistical ability to perform detailed subgroup analyses or detect subtle differences in recovery patterns. Third, due to the retrospective design of this study, total analgesic consumption was evaluated based on the frequency of rescue analgesic administration rather than cumulative oral morphine equivalent (OME) doses, which limits the granularity of our analgesic utilization data. Fourth, the use of a needle-prick AST VAS score as a surrogate for general surgical pain sensitivity is a novel and unvalidated method. A superficial, acute nociceptive response (such as a needle prick) may not accurately reflect a patient's tolerance for deep somatic or osteogenic surgical pain. Consequently, our attempt to control for inherent differences in baseline pain tolerance relies on an unvalidated proxy measure, and true baseline pain sensitivities may have varied between groups. Fifth, the retrospective design inherently limits our ability to establish definitive causation because we could not control for unmeasured intraoperative surgical and technical confounders. The intraoperative management of an LHF often requires additional surgical steps. Factors such as variations in total tourniquet time, the extent of soft tissue dissection or periosteal stripping, the application of bone grafts, and the extra operative time required for extended plate fixation may independently contribute to the observed elevation in early postoperative pain, regardless of the fracture itself. Finally, as a single-institution study, our findings reflect our specific institutional postoperative pain management protocol. Because different hospitals may employ varied analgesic strategies, these findings may not be fully generalizable to other clinical settings. Future multi-center studies are recommended to validate these results across diverse pain management environments.

## Conclusions

Intraoperative LHFs during MOWHTO are associated with a statistically significant increase in early postoperative pain. However, because this elevation (0.52 points) remains below the established MCID, the added clinical burden on the patient is negligible when managed appropriately. This study validates the efficacy of a comprehensive multimodal analgesic protocol, demonstrating its ability to successfully neutralize the excess pain associated with LHFs. Consequently, surgeons can confidently maintain their accelerated postoperative rehabilitation timelines following an intraoperative LHF, provided a robust pain management system is actively in place.
